# Supplementary material for: Neuronal parts list and wiring diagram for a visual system
Source: Nature. 2024 Oct 2;634(8032):166–80. doi: 10.1038/s41586-024-07981-1 (PMC11446827; doi:10.1038/s41586-024-07981-1)
Supplement: Supplementary file 5 — Cards for all cell types. Each card has 6 panels (clockwise): logical connectivity predicate diagram, numeric stats, illustration of a typical cell, followed by 3D rendering of all cells from three orthogonal viewpoints. Also available at Codex. [file 41586_2024_7981_MOESM5_ESM.zip › pdf/Tm8b.pdf]

Tm8b

predicate precision: 98%, recall: 92%

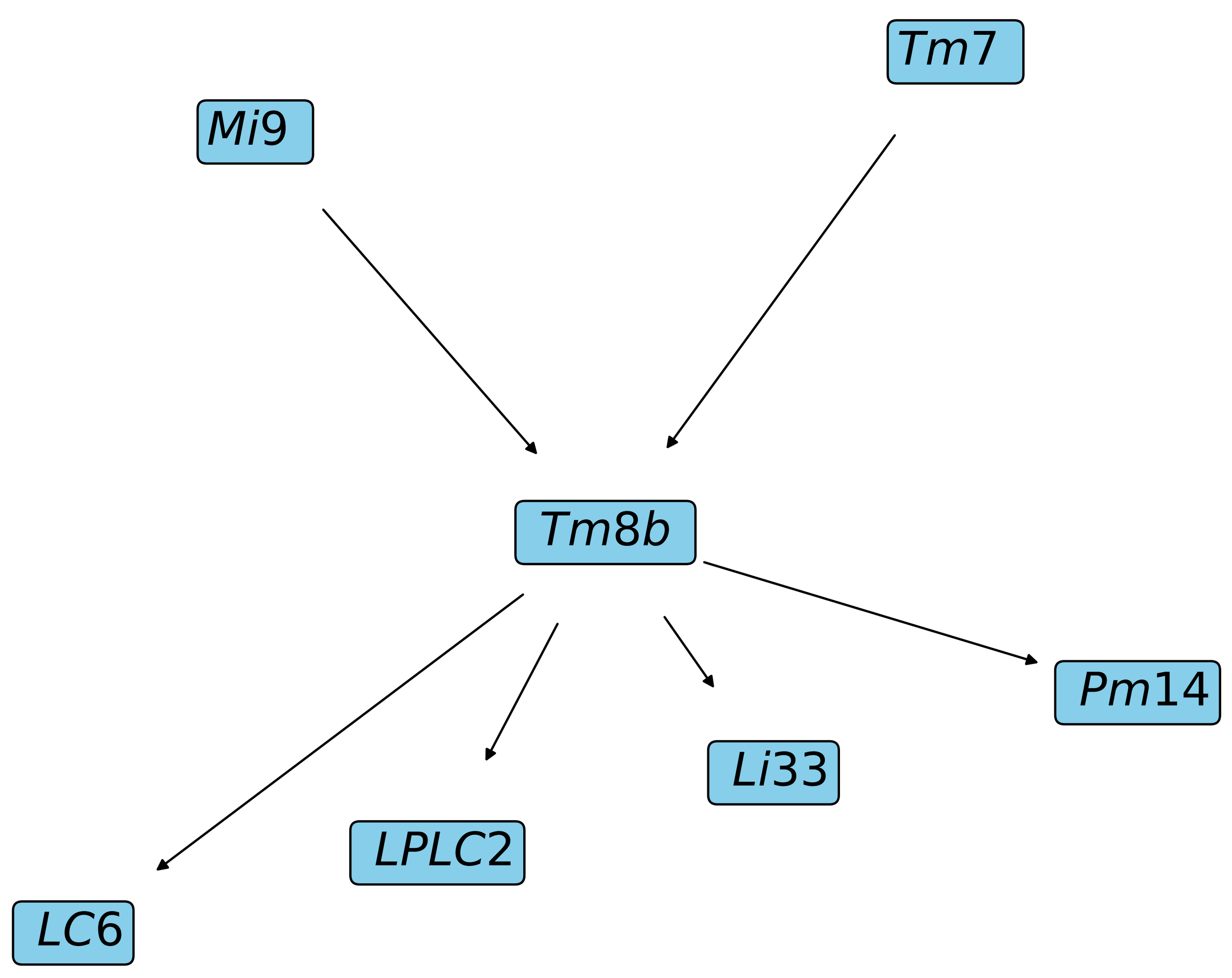

avg. stats

cells	59
NT prediction	ACH (100%)
top in/out regions	ME (86%) / LO (88%)
in/out degree	64 / 49
in/out synapses	238 / 227
cable len	930μ
surface	$1,326\mu^2$
volume	$76\mu^3$

morphology

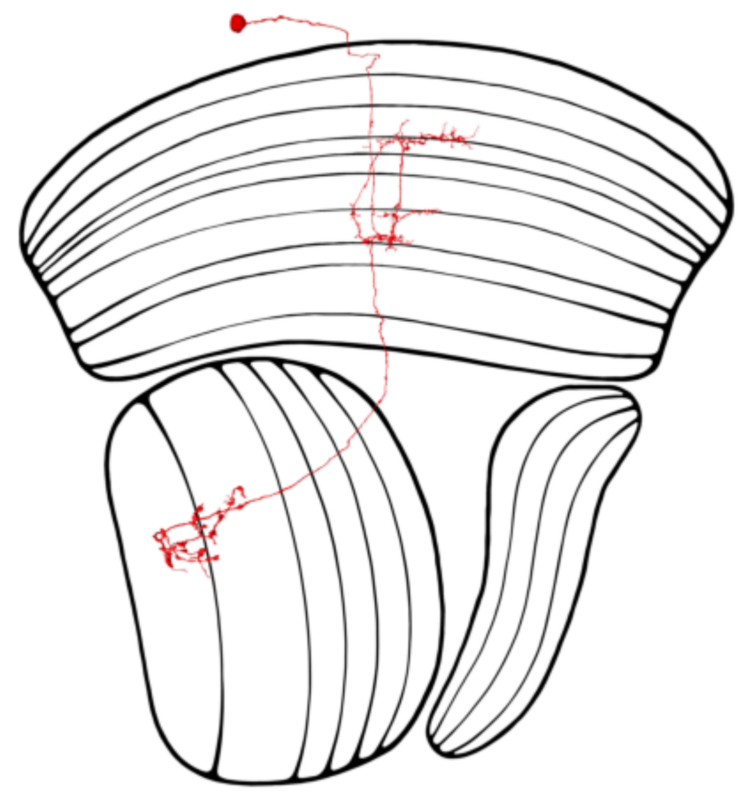

anterior view

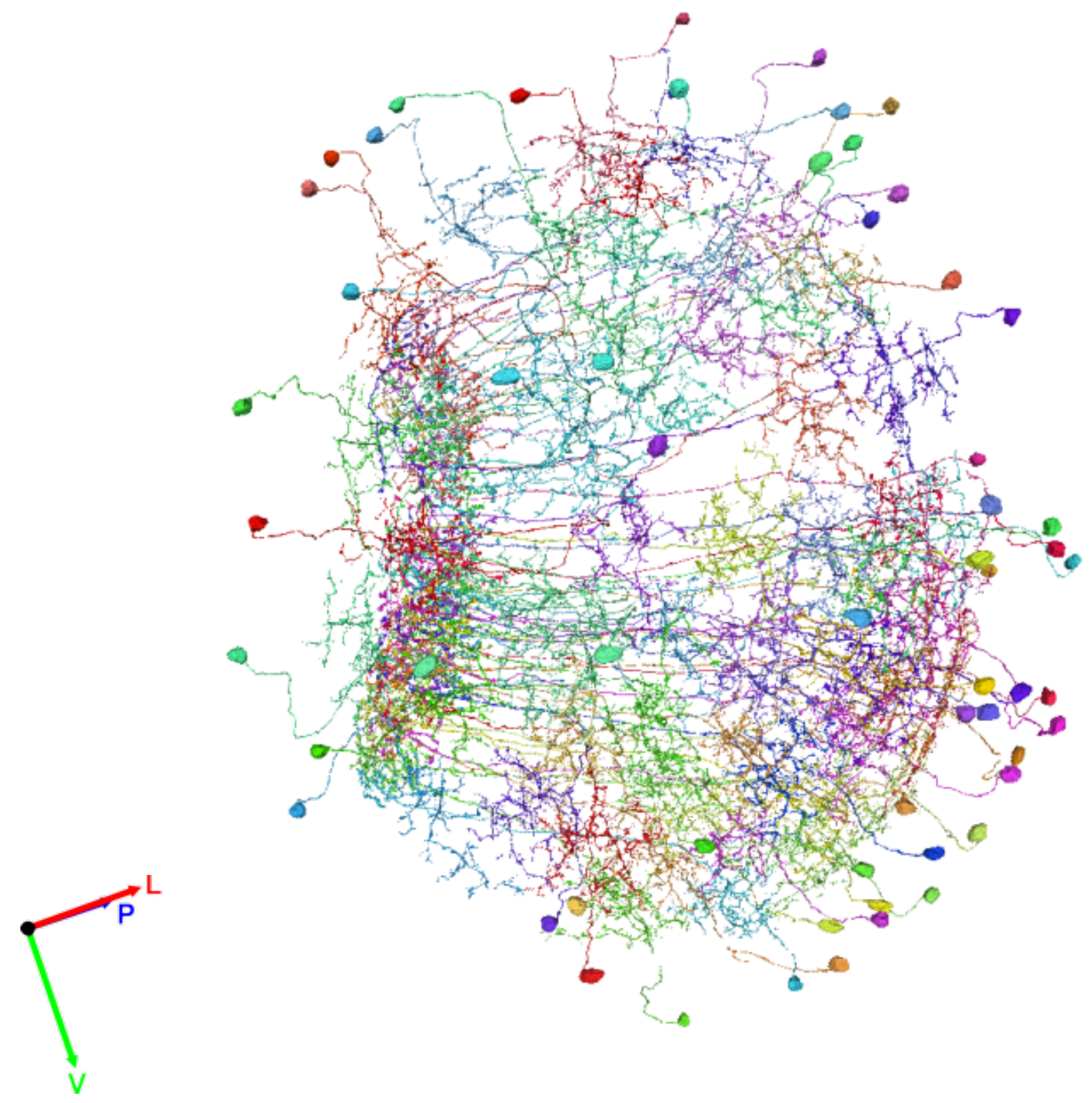

ventral view

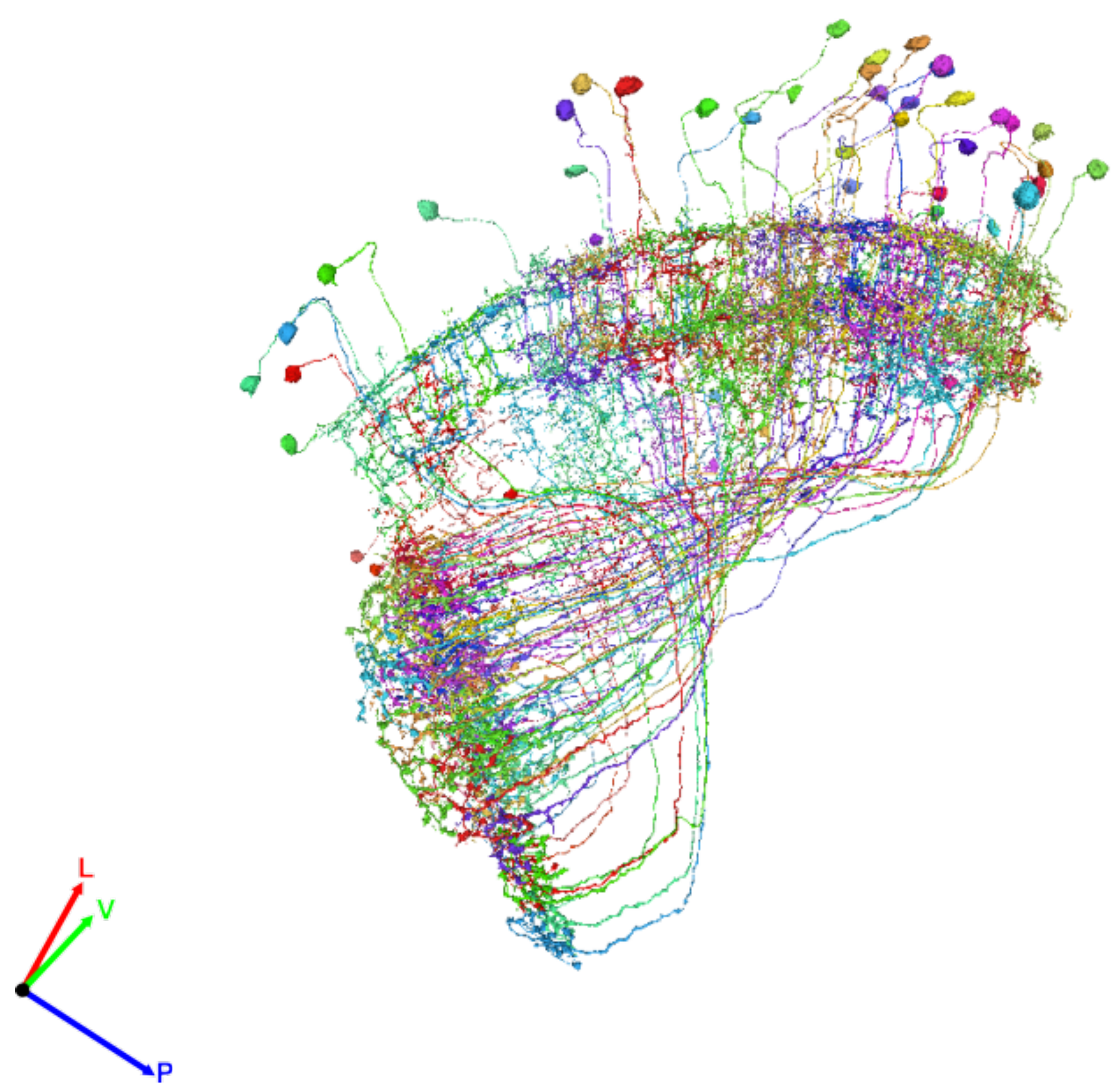

side view

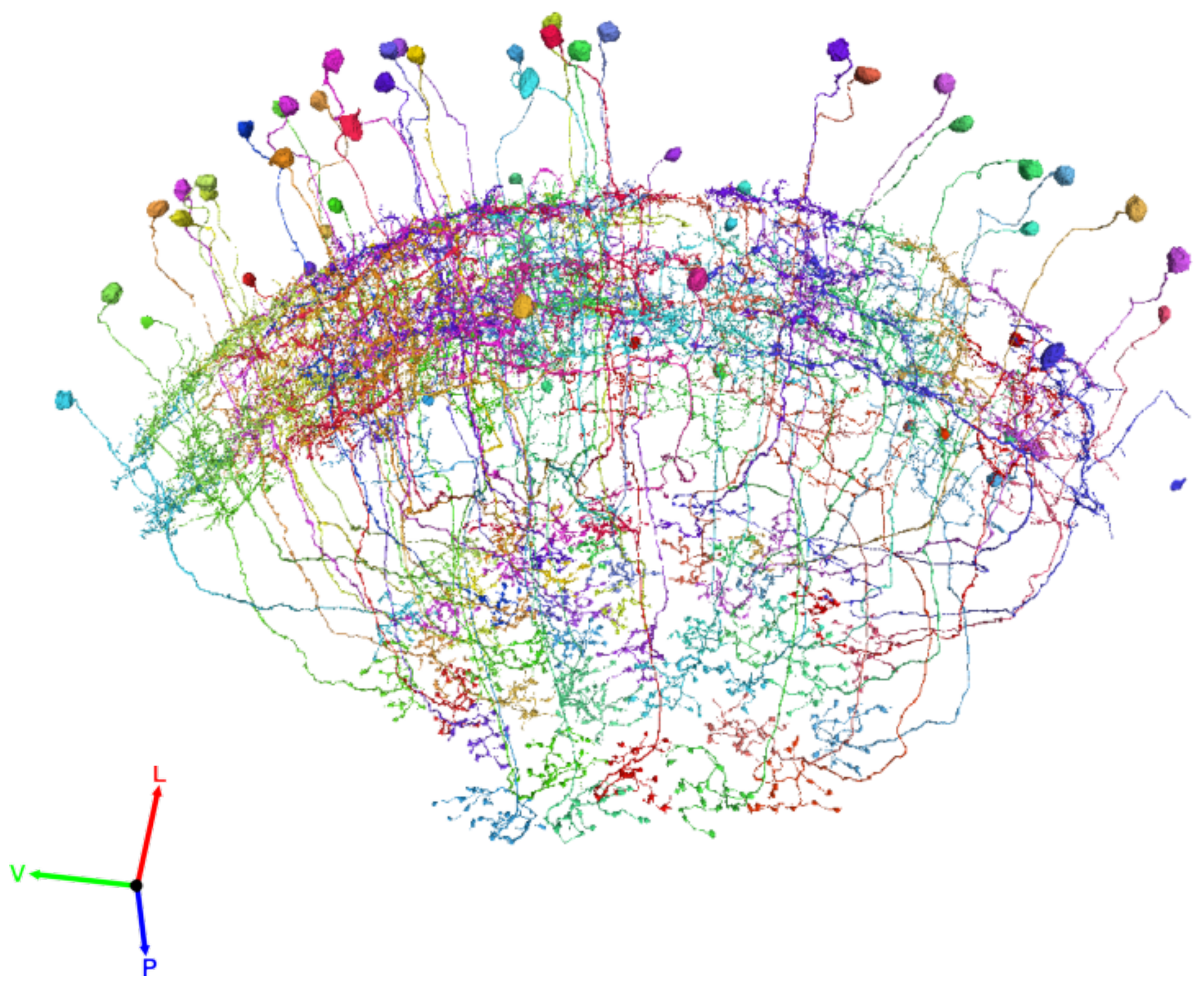

Transmedullary
